# Cost-effectiveness of 13-valent pneumococcal conjugate vaccination in Mongolia

**DOI:** 10.1016/j.vaccine.2016.12.070

**Published:** 2017-02-15

**Authors:** Neisha Sundaram, Cynthia Chen, Joanne Yoong, Munkh-Erdene Luvsan, Kimberley Fox, Amarzaya Sarankhuu, Sophie La Vincente, Mark Jit

**Affiliations:** a Saw Swee Hock School of Public Health, National University of Singapore, 12 Science Drive 2, #10-01, Singapore 117549, Singapore; b Mongolian National University of Medical Sciences, S. Zorig St-3, P.O. Box 48/111, Ulaanbaatar 14210, Mongolia; c Expanded Programme on Immunization, World Health Organization Regional Office for the Western Pacific, United Nations Ave Corner Taft Ave, Manila 1000, Philippines; d Ministry of Health and Sports, Government Building VIII, Olympic Street 2, Sukhbaatar District, Ulaanbaatar 14210, Mongolia; e Pneumococcal and International Child Health Research Groups, Murdoch Childrens Research Institute, Royal Children's Hospital, Flemington Road, Parkville, VIC 3052, Australia; f Department of Paediatrics, University of Melbourne, Royal Children's Hospital, Flemington Road, Parkville, VIC, Australia; g Modelling and Economics Unit, Public Health England, 61 Colindale Avenue, London NW9 5EQ, United Kingdom; h Department of Infectious Disease Epidemiology, London School of Hygiene and Tropical Medicine, Keppel Street, London WC1E 7HT, United Kingdom

**Keywords:** Pneumococcal conjugate vaccine, Cost-effectiveness, Mongolia, Vaccine, Budget impact, PCV13, AMC, advance market commitment, AOM, acute otitis media, CFR, case-fatality risk, DALY, disability-adjusted life year, GDP, gross domestic product, GNI, gross national income, Hib, haemophilus influenzae type b, ICER, incremental cost-effectiveness ratio, IPD, invasive pneumococcal disease, MNT, Mongolian tugrik, MOH, Ministry of Health, NPNM, non-pneumonia non-meningitis, OOP, out-of-pocket, PAHO, Pan American Health Organization, PCV, pneumococcal conjugate vaccine, WPR, Western Pacific region

## Abstract

**Objective:**

The Ministry of Health (MOH), Mongolia, is considering introducing 13-valent pneumococcal conjugate vaccine (PCV13) in its national immunization programme to prevent the burden of disease caused by *Streptococcus pneumoniae*. This study evaluates the cost-effectiveness and budget impact of introducing PCV13 compared to no PCV vaccination in Mongolia.

**Methods:**

The incremental cost-effectiveness ratio (ICER) of introducing PCV13 compared to no PCV vaccination was assessed using an age-stratified static multiple cohort model. The risk of various clinical presentations of pneumococcal disease (meningitis, pneumonia, non-meningitis non-pneumonia invasive pneumococcal disease and acute otitis media) at all ages for thirty birth cohorts was assessed. The analysis considered both health system and societal perspectives. A 3 + 0 vaccine schedule and price of US$3.30 per dose was assumed for the baseline scenario based on Gavi, the Vaccine Alliance’s advance market commitment tail price.

**Results:**

The ICER of PCV13 introduction is estimated at US$52 per disability-adjusted life year (DALY) averted (health system perspective), and cost-saving (societal perspective). Although indirect effects of PCV have been well-documented, a conservative scenario that does not consider indirect effects estimated PCV13 introduction to cost US$79 per DALY averted (health system perspective), and US$19 per DALY averted (societal perspective). Vaccination with PCV13 is expected to cost around US$920,000 in 2016, and thereafter US$820,000 every year. The programme is likely to reduce direct disease-related costs to MOH by US$440,000 in the first year, increasing to US$510,000 by 2025.

**Conclusion:**

Introducing PCV13 as part of Mongolia’s national programme appears to be highly cost-effective when compared to no vaccination and cost-saving from a societal perspective at vaccine purchase prices offered through Gavi. Notwithstanding uncertainties around some parameters, cost-effectiveness of PCV introduction for Mongolia remains robust over a range of conservative scenarios. Availability of high-quality national data would improve future economic analyses for vaccine introduction.

## 1 Background

*Streptococcus pneumoniae* caused an estimated 500,000 deaths worldwide among children under five years of age in 2008 [1]. In Mongolia, pneumonia is a leading cause of childhood mortality, as well as accounting for 51% of all-age respiratory disease admissions [2]. Severe air pollution, especially in winter, exacerbates the problems caused by pneumonia and other acute respiratory infections [3]. Shortage of equipment, drugs and skilled health professionals, mostly in rural areas, further contribute to high preventable mortality from pneumonia [4]. *S. pneumoniae* and *Haemophilus influenzae* type b (Hib) are leading causes of childhood pneumonia-related deaths [5] and cause a substantial portion of meningitis and sepsis, in the absence of vaccination. Since introduction of Hib vaccine in 2005 in Mongolia [6], the continuing high rate of childhood pneumonia is attributable to pneumococcus more than any other single cause.

In accordance with World Health Organization (WHO) recommendations to include pneumococcal conjugate vaccines (PCVs) in childhood immunization programs worldwide [1], Mongolia plans to introduce the 13-valent PCV (PCV13) into its Expanded Programme on Immunisation in a 3 + 0 schedule (three primary doses at 2, 3 and 4 months of age without a booster dose) that would coincide with the oral polio and pentavalent vaccine schedules [7]. PCV13 (or Prevnar-13®) protects against invasive pneumococcal disease (IPD), pneumonia and acute otitis media (AOM) from *S. pneumonia*e.

The pneumococcal Advance Market Commitment (AMC) is an innovative funding mechanism incentivising vaccine makers to produce affordable vaccines for the world's poorest countries. Ministry of Health (MOH), Mongolia applied to Gavi, the Vaccine Alliance (Gavi) in 2013 to purchase PCV13 through the AMC mechanism and received Gavi approval in March 2014 for PCV introduction starting in 2016, with the intent of scaling up to a nationwide programme. Although Mongolia’s current Gross National Income (GNI) per capita is above Gavi’s threshold and the country is therefore transitioned out of Gavi support in 2016, it remains eligible for PCV vaccine prices under the AMC even after being fully self-financed [8]. However, introduction of PCV will still require financing to cover costs of vaccine purchase and vaccination within the immunization programme. Mongolia’s government has thus identified the need to assess the cost-effectiveness and financial sustainability of PCV13 introduction in the Mongolian context [7].

A collaboration between MOH, local and foreign investigators was established to conduct an economic evaluation for PCV introduction to inform decision making and establish the case for sustained investment. This evaluation is the first country-specific study to assess the costs and outcomes associated with PCV13, in order to determine whether PCV13 is cost-effective to introduce as part of Mongolia’s national immunisation programme, as well as its likely budget impact.

## 2 Methods

### 2.1 Model overview and analytic framework

**Fig. 1:**
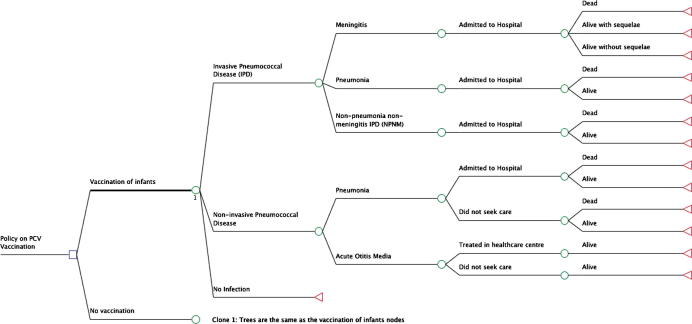
Decision tree for a single year of age, depicting two alternative strategies: vaccination with PCV-13 versus no vaccination. An age-stratified decision tree economic model to represent disease outcomes and associated health states for vaccinated and unvaccinated populations. The same structure is repeated for every year of age between 0 and 100 years. The ‘no vaccination’ node has the same branches as the vaccination node.

In order to investigate the value for money and financial sustainability respectively of vaccination the incremental cost-effectiveness of introducing PCV13 compared to no PCV vaccination was assessed using an age-stratified static multiple-cohort model (Fig. 1). The model assesses the risk of various clinical presentations of pneumococcal disease—meningitis (including sequelae), pneumonia, non-pneumonia non-meningitis (NPNM) IPD and AOM)—for each year of life between 0 and 100, in both vaccinated and unvaccinated individuals, and both with and without a vaccination programme. Each disease episode is associated with a cost and health utility loss. Thirty consecutive birth cohorts were assessed over a thirty year time period. The direct effect of PCV (direct population effects) is assessed by a proportionate reduction in pneumococcal disease risk in vaccinated individuals. The indirect effects of PCV introduction—herd protection (referring to a lower risk of infection among unvaccinated individuals due to increase in population-level immunity, generated by reduction in carriage of vaccine serotypes) and serotype replacement (a phenomenon referring to an increase in incidence of invasive disease caused by non-vaccine serotypes and proportion of carriage of non-vaccine serotypes after vaccine introduction)—are also considered by an adjustment to disease risk in unvaccinated individuals in the presence of a population-wide vaccination programme. Although there is substantial evidence for the existence of these indirect effects from post-introduction surveillance [9], [10], the magnitude of such effects is uncertain, so we considered an alternative conservative scenario without indirect effects.

The budget impact analysis was conducted over a 10 year horizon. For each year of the analysis, costs were calculated from net costs from all modelled birth cohorts (including adult cohorts affected by indirect vaccine effects) that were born in the same or previous years. All direct costs were assumed to be included in the budget impact; indirect societal costs were also shown in a sensitivity analysis.

Costs were inflated to 2014 prices based on Mongolia’s inflation rate of 15.0% in 2012 and 8.6% in 2013 [11]. Costs were then converted into United States (US) dollars using the average exchange rate for the year 2014 between the US dollar (US$) and the Mongolian tugrik (MNT): 1 US$ = 1804.50 MNT. Future costs and outcomes were discounted from the first year of vaccination at 3% per annum [12]. The analysis was done from the perspective of both the health system and society. Health system costs included vaccine costs (purchase, freight and administration), cold chain, surveillance, and hospitalization or health centre consultation costs. Societal costs considered productivity losses and out-of-pocket expenses in addition to health system costs noted above. Table 1 shows base case parameters used in the model.

### 2.2 Demographics

Population estimates for 2012 stratified by year of age were obtained in hard copy from the National Statistical Office, Mongolia and life expectancies were obtained from WHO’s 2011 Life Tables for Mongolia [13].

### 2.3 Vaccine coverage

A 3 + 0 schedule with vaccine coverage of 98.2% and 97.6% for first and third dose, respectively, was assumed, based on administrative coverage data of diphtheria-tetanus-pertussis vaccine given at the same ages [14]. Second dose coverage was assumed to be the average of the first and third doses. In addition, buffer stock of 25% of first dose coverage and 2% wastage (based on wastage for pentavalent vaccine communicated through the Expanded Programme on Immunization) was assumed.

### 2.4 Vaccine cost

A per-dose cost of $3.30 was used, since Mongolia is eligible to purchase pneumococcal vaccines at the Gavi AMC ‘tail price’ [8] set at $3.30 from 2014 onwards based on the third AMC supply agreement (22 July 2013) [15]. An additional 4% was added to account for vaccine freight as well as $0.0605 and $0.008 per dose for syringe and safety box purchase, respectively [16]. Customs and handling costs at 19% were included [16]. The cost of 5 min of a nurse’s time $229.27/month (413,720 MNT/month) based on a nurse’s average salary was further assumed.

Cold chain costs included purchases of refrigerators (at approximately $1000 per unit) to store vaccine doses at the central level and in the provinces based on a quarterly procurement of PCV13 [17]. Surveillance for pneumococcal disease has already been established in Mongolia, so only incremental annual maintenance costs of $100,738 were included to cover costs for centralized specimen testing and data management, based on past WHO-funded surveillance activities.

### 2.5 Vaccine efficacy

Efficacy of 9-valent PCV with a 3 + 0 schedule in HIV negative patients from a South African trial (83%) [18] was used as HIV prevalence in Mongolia is very low [19]. Efficacy data from a study in Gambia were not used as they were not stratified by HIV status [20]. However, PCV13 has shown poor efficacy against IPD associated with serotype 3, ranging from 68% in the USA [21] to non-significant in England [22]. Hence, an overall efficacy of 34% against serotype 3 IPD was assumed. We also assumed that 1.03% of vaccine type IPD was caused by serotype 3 based on estimates for Asia [23]. Efficacy against vaccine type IPD was thus scaled down to 82.5%. Vaccine efficacy of 20.2% against all-cause AOM was assumed [24], by scaling up PCV10 vaccine efficacy of 19% in the most recent trial (COMPAS) [24] to include the contribution of PCV13 against serotypes 6A and 19A. Average vaccine duration of protection of 8.3 years was assumed as with previous modelling work [25]. It was conservatively assumed that three doses were required for protection.

Distribution of serotypes was based on the Pneumococcal Global Serotype Project, where proportion of IPD due to each serotype for Asia were used (73.7%) [23]. No reliable carriage data for Mongolia were available, so we instead used data from a study of nasopharyngeal swabs of children visiting outpatient clinics in Kazakhstan, Uzbekistan and Kyrgyz Republic [26].

### 2.6 Disease burden

Incidence and case-fatality risks (CFRs) for pneumococcal meningitis, NPNM IPD and pneumonia was obtained from WHO estimates for Mongolia [27]. These are summarised in Table 1; details of their estimation are in Supplementary material. Mongolia estimates were scaled by data from Philippines [28] and rural Thailand [29] for age-specific incidences. All-cause AOM incidence was obtained from a systematic review of published studies [30].

### 2.7 Hospitalization and health centre visit costs, out-of-pocket costs and productivity losses

Hospitalization costs were obtained from sources in Mongolia and are summarised in Table 1 with details in Supplementary material. Similarly for parameters of medical care-seeking for children with pneumonia and AOM [31], [32].

To calculate productivity losses for caregivers of children under 18 years old, the average hospital stay or health centre visit was multiplied by the female labour force participation rate (58.4%) [33]. Each day of work lost was valued at Mongolia’s gross domestic product (GDP) per capita [34]. Out-of-pocket (OOP) expenditures were estimated using the WHO global health expenditure database [35] and a local pneumonia costing study. They included expenses for travel, additional drugs and tests and miscellaneous expenses as determined through patient surveys. They are summarised in Table 1 with details in Supplementary material.

### 2.8 Disability weights

Disability-adjusted life years (DALYs) lost due to non-fatal pneumococcal meningitis, pneumonia, NPNM IPD and AOM were obtained from the Global Burden of Disease [36] and the risk of different kinds of major sequelae of pneumococcal meningitis was obtained from a global meta-analysis [37], adjusted for WHO Western Pacific region (WPR). Further details of their estimation are in Supplementary material.

### 2.9 Indirect effects

Indirect effects were extrapolated from IPD incidence following PCV13 introduction in the United Kingdom (UK) between 2010/11 and 2013/14 [38]. Based on UK observed data, age-dependent incidence prior to PCV7 introduction (2005/2006) was used as a baseline and proportional decrease in yearly age-dependent incidence from 2010 to 2014 were calculated. This proportional decline was applied to Mongolian incidence in the first four years of PCV introduction and we assumed constant proportion thereafter. Post-introduction surveillance in other countries [9], [39] show the same qualitative trends, but UK data was used because it tracks the year-on-year change from baseline. Changes in incidence of vaccine-type and non-vaccine type IPD compared to the last year before vaccine introduction (2006/7) were used as indications of herd protection and serotype replacement, respectively. The direct effect of vaccination on vaccine-type disease was estimated as the product of vaccine efficacy for a 2 + 1 schedule in a high-income context (95%) [25] and coverage (95%) in vaccinated age groups. It was assumed that in the first year effective vaccine coverage is halved due to the time it takes to vaccinate an entire age cohort. The proportionate decrease in vaccine-type IPD and increase in non-vaccine type IPD was then applied to Mongolia, after adjusting proportionally for differences in vaccine coverage, serotype distribution and efficacy of the 3 + 0 schedule in a middle-income context. In the base case, given 97.6% coverage for 3 doses, 73.7% serotype coverage and 82.5% vaccine efficacy, indirect effects were assumed to be 88.6% of that in the UK.

### 2.10 Sensitivity analyses

To explore the robustness of conclusions to assumptions, the following sensitivity scenarios were modelled by varying costs and disease burden (Table 2):

i. *Vaccine cost:* Scenarios with higher vaccine purchase cost (a) Gavi AMC maximum tail price of $3.50 [8] and (b) the higher Pan American Health Organization (PAHO) Revolving Fund price of $15.68 [40]
ii. *Hospitalization cost:* As an upper limit for potential hospitalization costs, in the event of escalating costs, we include a scenario using outputs from WHO-CHOICE to estimate hospitalization cost for pneumococcal meningitis, pneumonia or NPNM IPD. In a univariate linear regression model using the sample of available countries, GNI per capita significantly predicted average treatment cost for meningitis and pneumonia [41], with a reasonable model fit (R^2^ of 83% for meningitis and 72% for pneumonia). Applying the regression estimates to 2013 GNI per capita for Mongolia ($3,770) [42], we estimated the average annual costs per hospitalized case for meningitis and pneumonia to be $1,771 and $528. NPNM IPD treatment cost was assumed to be the same as for meningitis. Out-of-pocket expenditures were calculated as before using a factor of 35% of Total Health Expenditure [35].
iii. *Disease burden:* A minimum-impact scenario for vaccine introduction with low disease rates and CFR, using lower bounds from WPR estimates, and conversely a maximum-impact scenario using upper bounds from WPR estimates were considered [43]. Regional lower and upper bound estimates per 100,000 were: pneumonia: 1381–2208; meningitis: 3–14; and NPNM IPD: 17–84 [43]. In all cases, incidence in the base case scenario was similar to the regional estimate or its lower bound. Lower and upper bounds for regional CFR estimates were: meningitis: 10–68%, pneumonia: 1–3% and severe NPNM IPD: 8–53% [43].

## 3 Results

### 3.1 Base case

The incremental cost-effectiveness ratio (ICER) per DALY averted from PCV13 introduction in Mongolia is estimated to be $52 from a health system perspective, and cost-saving from a societal perspective (Table 3). In an alternative conservative scenario that considers population direct effects only without herd effects due to reduced transmission (although herd effects of pneumococcal conjugate vaccines have been well-documented), introducing PCV13 is estimated to cost $79 from a health system perspective, and $19 from a societal perspective (Table 3).

### 3.2 Sensitivity analyses

Table 4 shows how the cost per DALY averted from PCV13 vaccination may change for the scenarios considered in the sensitivity analyses. The most pessimistic scenario was purchase of PCV13 at the higher PAHO price, which cost $460 and $390 per DALY averted from health system and societal perspectives, respectively ($540 and $480, respectively, when considering the unlikely but further conservative case of population direct effects only from vaccine). The minimum impact scenario that considered the conservative lower bounds of disease incidence and CFRs, cost $110 per DALY averted from a health system perspective and was cost-saving from a societal perspective. Additional, one-way sensitivity analyses varying vaccine-related parameters, disease burden and disability weights by ±25% also showed PCV13 vaccination to be cost-effective (Tornado diagram, see Supplementary figure).

### 3.3 Budget impact

**Fig. 2:**
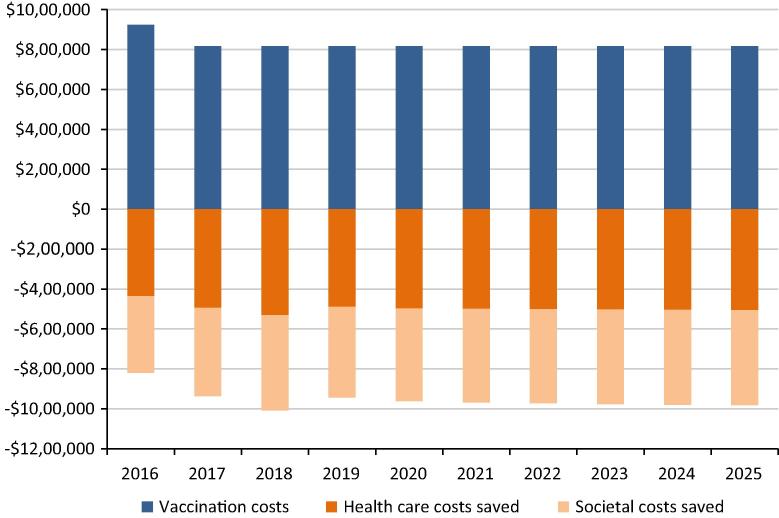
Budget impact of PCV13 vaccination in Mongolia over 2016–2025. Undiscounted vaccination costs (at $3.3 per dose based on purchase price for Ministry of Health, Mongolia through Gavi, the Vaccine Alliance’s AMC tail price) and costs saved from both a health system perspective as well as societal perspective are displayed.

Fig. 2 displays the impact that vaccination with PCV13 may have on the health care budget for MOH, as well as on wider societal costs, in 2014 US$ (i.e., not considering inflation). A constant birth cohort size and no vaccine price maturation were assumed. Vaccination with PCV13 is expected to cost around $920,000 in the first year assuming increase in cold chain capacity, and thereafter cost around $820,000 every year. The vaccination programme is likely to reduce direct costs to the health care budget by about $440,000 in the first year, increasing to $510,000 by 2025. Societal costs, which includes productivity losses and out-of-pocket expenses, are also likely to be reduced by about $380,000 in 2016, rising to $480,000 by 2025.

## 4 Discussion

The ICER for PCV13 introduction in Mongolia—$52 per DALY averted and cost-saving from health care provider and societal perspectives, respectively—is substantially lower than Mongolia’s GDP per capita ($4,056.40 in 2013) sometimes used as a threshold for cost-effectiveness [34], as well as a much lower threshold of $122–$173 suggested by the University of York based on the mortality effects of health expenditure [44]. It also compares favourably to previously estimated ICERs of $114–$123 per DALY for rotavirus vaccination [45] and $470 per DALY for HPV vaccination at Gavi prices [46]; and $91–$110 per DALY from a regional analysis for Hib vaccine [41]. Hence PCV13 introduction is likely to be a cost-effective decision for Mongolia. The ICER of PCV13 introduction remains well below GDP per capita (and usually below the other thresholds examined) across the range of potential scenarios considered.

Our study is the first country-specific PCV cost-effectiveness evaluation in Mongolia, but is consistent with more general analyses. For example, one study concluded that using PCV in the 72 Gavi-eligible countries in 2005 would have an ICER of about $100 per DALY prevented; this analysis used the societal perspective and considered direct population effects only [47]. The corresponding ICER from our analysis for Mongolia was $19. While care should be taken in comparing results from economic models, this suggests that PCV13 is indeed highly cost-effective for Mongolia.

The price at which PCV13 is being offered through Gavi is a major factor in making PCV13 highly cost-effective or cost-saving for introduction in Mongolia. Although the price is low relative to prices offered for PCV13 globally, the total vaccine cost to the country still amounts to $920,000 in the first year due to additional cold chain capacity costs for PCV and $820,000 per year in subsequent years for national introduction. Furthermore, even at the higher PAHO revolving fund price, which we do not reasonably expect Mongolia’s vaccine purchase price to exceed even after transition out of Gavi’s AMC price, PCV13 introduction was highly cost-effective. In addition to vaccine cost, disease incidence was a major driver influencing the cost-effectiveness analysis. However, even at lower bounds of incidence and case fatality risk, introduction of PCV13 was cost-effective for Mongolia.

Mongolian public expenditure on health in 2014 is 2.6% of GDP, which is around three hundred million dollars. As seen from the budget impact analysis, total yearly PCV13 vaccine costs to the country represents approximately 0.3% of the annual public spending on health. While this is not trivial sum, a decreased pneumococcal disease burden from PCV vaccination is estimated to reduce disease-related spending by $500,000. Furthermore, when economic costs to the wider society are considered, additional savings of $400,000 per year results in an annual potential budget saving of around $100,000 to $200,000.

A limitation of this analysis was an absence of local data for age-specific disease burden, as is common in many low and middle income countries with limited resources for bacteriological surveillance. Data from the Philippines and Thailand was thus extrapolated based on inter-country similarities in terms of GNI per capita, life expectancy, proportion of deaths from and health-seeking for respiratory infections, etc. (see Supplementary material. Country profiles). Availability of local data would enhance the representativeness of this analysis. However, our results are robust to varying the parameters extrapolated from other settings within large ranges representing the range of values of countries in the WPR. Hence our analysis suggests ways that useful conclusions can be drawn from economic evaluations in countries with limited epidemiological data. More local data would also allow us to conduct probabilistic sensitivity analyses to assess uncertainty of all parameters together.

Finally, some assumptions were made regarding the health system’s capacity for effective delivery of this additional vaccine including necessary investments to maintain the quality of surveillance for adverse events following immunization, supply distribution systems and monitoring. Furthermore, other healthcare costs, such as training, social mobilization and surveillance capacity building prior to vaccine introduction were not included. Our model therefore underestimates the administrative costs of vaccine introduction. However, these costs are expected to be small relative to the costs of vaccine and related supplies.

Recently, MOH and WHO in Mongolia have discussed using a 2 + 1 vaccine schedule instead of the originally planned 3 + 0 schedule [7], which this analysis is based on. Since, number of vaccine doses remains unchanged and no significant differences in impact on disease burden between schedules have been currently established [48], [49], we do not expect the conclusions to change for a 2 + 1 schedule.

In conclusion, routine infant vaccination against *S. pneumoniae* with PCV13 appears to be highly cost-effective when compared to no vaccination in Mongolia. Continued investment in this vaccination programme is likely to be the right economic decision despite the present financial challenges in Mongolia.

## Conflict of interest

The authors have no conflict of interest to declare.

## Figures and Tables

**Table 1:** Base case parameters used in the model.

Parameter	Value	Source
Vaccine characteristics		
*Efficacy*		
… against vaccine-type IPD	82.5%	Klugman 2003 [18] Kaplan 2013 [21] and Andrews 2014 [22]
… against vaccine-type pneumococcal carriage	82.5%	Assumed same as vaccine-type IPD
… against all-cause acute otitis media (PCV13)	20.2%	Tregnaghi 2014 [24]

*Coverage (in targeted population)*		
… dose one	98.2%	Ministry of Health, Mongolia. WHO/UNICEF Joint Reporting Form on Immunization, data for 2013 [14]
… dose two	97.9%	Estimated as average of first and third doses
… dose three	97.6%	Ministry of Health, Mongolia. WHO/UNICEF Joint Reporting Form on Immunization, data for 2013 [14]

Costs (US$2014)		
*Vaccination*		
… purchase (PCV13)	$3.30	Gavi, the Vaccine Alliance [15]
… freight (PCV13)	$0.14	UNICEF Supply Division, Immunization forecast overview, Recommended budget prices for 2014 [16]
… buffer stock	25%	Assumption

*Hospitalization*		
… meningitis	$271.0	Health Insurance Fund, Mongolia
… pneumonia	$160.3	Pneumonia costing study 2012, Mongolia
… invasive NPNM	$271.0	Same as for meningitis

*Health centre consultation*		
… acute otitis media	$8.3	Decree by Ministry of Health and Social Health Insurance Office, Mongolia in 2014

*Productivity costs*		
…female labour force participation rate	58.4%	National Statistical Office of Mongolia, Ulaanbaatar 2013 [33]
…Gross domestic product per capita in 2014	$4056.4	World Bank, 2014. World Development Indicators. [34]
…Meningitis average hospital stay	10 days	2012 hospital records matched by ICD-10 codes from National Center for Health Development, Mongolia
…Sepsis (proxy for NPNM) average hospital stay	9 days	
…Pneumonia average hospital stay	7 days	
…Days of work lost from acute otitis media	1 day	Assumption

*Out of pocket (OOP) costs*		
… meningitis	$145.9	OOP as percentage of total health care expenditure [35]
… pneumonia	$86.3	Pneumonia costing study 2012, Mongolia
… invasive NPNM	$145.9	OOP as percentage of total health care expenditure [35]
… acute otitis media	$4.5	OOP as percentage of total health care expenditure [35]

*Proportion of patients who seek care*		
… Acute otitis media (health care centre)	48%	Kazakhstan DHS,1999 [32]
… Non-invasive pneumonia	87%	National Statistics Office, UNICEF. MICS 2010 Summary Report [31]
Disease burden		

*Incidence (per 100,000)*		
…meningitis	11	WHO estimate [27], scaled by age-specific incidence from Capeding 2013 [28]
…invasive NPNM	62	
…pneumonia	1345	WHO estimate [27], scaled by age-specific incidence from Hasan 2014 [29]

*Case-fatality risk*		
…meningitis	34.6%	WHO estimate for Mongolia [27]
…invasive NPNM	27.1%	WHO estimate for Mongolia [27]
…pneumonia	5.4%	WHO estimate for Mongolia [27]

Disability weights		
… Meningitis (per episode)	0.62	“Meningitis, S. pneumoniae” in Mathers 2006 [36]
… Pneumonia (per episode)	0.28	“Neonatal pneumonia” in Mathers 2006 [36]
… NPNM (per episode)	0.15	“Meningococcaemia without meningitis” in Mathers 2006 [36]
… Otitis media (per episode)	0.02	“Otitis media” in Mathers 2006 [36]
… Major cognitive difficulties (per year)	0.46	“Mental retardation” in Mathers 2006 [36]
… Major seizure disorder (per year)	0.10	“Seizure” in Mathers 2006 [36]
… Major hearing loss (per year)	0.12	“Deafness” in Mathers 2006 [36]
… Major motor deficit (per year)	0.38	“Motor deficit” in Mathers 2006 [36]
… Major visual disturbance (per year)	0.60	“Blindness” in Mathers 2006 [36]
… Major clinical impairment (per year)	0.10	“Seizure” in Mathers 2006 [36]

*Pneumococcal meningitis sequelae risk*		
… Major cognitive difficulties	3.6%	Edmond [37]
… Major seizure disorder	3.0%	Edmond [37]
… Major hearing loss (per year)	8.0%	Edmond [37]
… Major motor deficit (per year)	3.9%	Edmond [37]
… Major visual disturbance (per year)	1.4%	Edmond [37]
… Major clinical impairment (per year)	4.0%	Edmond [37]

Costs were standardized for 2014 prices, and where necessary, inflated to 2014 prices based on Mongolia’s inflation rate of 15% in 2012 and 8.6% in 2013. IPD: invasive pneumococcal disease; nIPD: non-invasive pneumococcal disease; NPNM: non-pneumonia non-meningitis.

**Table 2:** Parameters for various sensitivity scenarios and base case.

	Base case	**S1:** Max Gavi tail $	**S2:** PAHO $	**S3:** WHO-CHOICE	**S4:** Low incid & CFR	**S5:** High incid & CFR
**Cost**						
*Vaccine cost*	**$3.30**	**$3.50**	**$15.68**	$3.30	$3.30	$3.30

*Hospitalization cost*						
Meningitis	**$271.02**	$271.02	$271.02	**$1,771.44**	$271.02	$271.02
Pneumonia	**$160.30**	$160.30	$160.30	**$527.63**	$160.30	$160.30
NPNM	**$271.02**	$271.02	$271.02	**$1,771.44**	$271.02	$271.02

*Out-of-pocket cost*						
Meningitis	**$145.93**	$145.93	$145.93	**$953.85**	$145.93	$145.93
Pneumonia	**$86.32**	$**86.32**	$**86.32**	**$284.11**	$**86.32**	$**86.32**
NPNM	**$145.93**	$145.93	$145.93	**$953.85**	$145.93	$145.93
AOM	**$4.48**	$4.48	$4.48	**$5**	$4.48	$4.48

**Incidence**						
*Meningitis*						
0 year old	**0.00060**	0.00060	0.00060	0.00060	**0.00003**	**0.00014**
1 year old	**0.00018**	0.00018	0.00018	0.00018	**0.00003**	**0.00014**
2 year old	**0.00004**	0.00004	0.00004	0.00004	**0.00003**	**0.00014**
3 year old	**0.00003**	0.00003	0.00003	0.00003	**0.00003**	**0.00014**
4 year old	**0.00006**	0.00006	0.00006	0.00006	**0.00003**	**0.00014**

*Pneumonia IPD*						
0 year old	**0.00063**	0.00063	0.00063	0.00063	**0.00065**	**0.00103**
1 year old	**0.00035**	0.00035	0.00035	0.00035	**0.00036**	**0.00058**
2 year old	**0.00035**	0.00035	0.00035	0.00035	**0.00036**	**0.00058**
3 year old	**0.00035**	0.00035	0.00035	0.00035	**0.00036**	**0.00058**
4 year old	**0.00035**	0.00035	0.00035	0.00035	**0.00036**	**0.00058**

*NPNM*						
0 year old	**0.00228**	0.00228	0.00228	0.00228	**0.00017**	**0.00084**
1 year old	**0.00178**	0.00178	0.00178	0.00178	**0.00017**	**0.00084**
2 year old	**0.00052**	0.00052	0.00052	0.00052	**0.00017**	**0.00084**
3 year old	**0.00012**	0.00012	0.00012	0.00012	**0.00017**	**0.00084**
4 year old	**0.00024**	0.00024	0.00024	0.00024	**0.00017**	**0.00084**

*Pneumonia nIPD*						
0 year old	**0.02038**	0.02038	0.02038	0.02038	**0.02092**	**0.03345**
1 year old	**0.01135**	0.01135	0.01135	0.01135	**0.01165**	**0.01863**
2 year old	**0.01135**	0.01135	0.01135	0.01135	**0.01165**	**0.01863**
3 year old	**0.01135**	0.01135	0.01135	0.01135	**0.01165**	**0.01863**
4 year old	**0.01135**	0.01135	0.01135	0.01135	**0.01165**	**0.01863**

**Case fatality risk**						
Meningitis U5	**0.346**	0.346	0.346	0.346	**0.100**	**0.680**
Pneumonia IPD U5	**0.054**	0.054	0.054	0.054	**0.010**	**0.030**
NPNM U5	**0.271**	0.271	0.271	0.271	**0.080**	**0.530**

S: scenario; CFR: case fatality risk; Incid: Incidence; U5: children under 5 years old; NPNM: non-pneumonia non-meningitis; IPD: invasive pneumococcal disease; nIPD: non-invasive pneumococcal disease.

S1: Higher vaccine cost-Gavi AMC maximum tail price; S2: Higher vaccine cost- Pan American Health Organization Revolving Fund price; S3:Higher hospitalization costs from WHO-CHOICE; S4: Minimum-impact scenario for vaccine introduction, using lower bounds from regional estimates for incidence and CFR for meningitis, pneumonia and NPNM IPD; S5: maximum-impact scenario from vaccine introduction, using upper bounds from regional estimates for incidence and CFR for meningitis, pneumonia and NPNM IPD.

All parameters of the base case and parameters in each of the scenarios where they differ from the base case have been highlighted in bold characters.

**Table 3:** Incremental outcomes of PCV13 compared to no vaccination over thirty years.

		Direct and indirect population effects (base case)^a^		Direct population effects only^b^	
	No vaccination with PCV	Vaccination with PCV13	Difference: Vaccination with PCV13 vs. no PCV vaccination	Vaccination with PCV13	Difference: Vaccination with PCV13 vs. no PCV vaccination
Number of children vaccinated with 3 doses	0	1.9 mil	1.9 mil	1.9 mil	1.9 mil
Total vaccination cost (undiscounted)	$0	$19 mil	$19 mil	$19 mil	$19 mil
Freight, administration cost (undiscounted)	$0	$5.6 mil	$5.6 mil	$5.6 mil	$5.6 mil
Health care costs (undiscounted)	$51.7 mil	$36.6 mil	−$15.1 mil	$40.0 mil	−$11.8 mil
Societal costs (undiscounted)	$54.6 mil	$40.5 mil	−$14.2 mil	$44.3 mil	−$10.4 mil
IPD cases	17,700	10,800	−6,890	10,040	−7,700
Non-IPD pneumonia cases	217,000	132,000	−85,200	158,000	−58,900
Acute otitis media cases	4,360,000	3,940,000	−417,000	3,940,000	−417,000
Deaths	15,200	9,240	−5,950	10,300	−4,900
DALYs lost (undiscounted)	548,000	364,000	−184,000	378,000	−170,000
DALYs lost (discounted)	358,000	237,000	−121,000	250,000	−108,000
Total costs with productivity (undiscounted)	$106 mil	$102 mil	−$4.72 mil	$109 mil	$2.44 mil
Total costs with productivity (discounted)	$ 69.5 mil	$66.5 mil	−$2.96 mil	$71.5 mil	$2.02 mil
Total costs without productivity (undiscounted)	$51.7 mil	$61.2 mil	$9.46 mil	$64.5 mil	$12.8 mil
Total costs without productivity (discounted)	$33.8 mil	$40.0 mil	$6.25 mil	$42.4 mil	$8.62 mil
Incremental cost-effectiveness ratio (with productivity)		Cost saving		$19	
Incremental cost-effectiveness ratio (without productivity)		$52		$79	

Mil: million; Incremental cost-effectiveness ratio: Cost per DALY averted; IPD: Invasive pneumococcal disease.

In this analysis, following World Health Organization recommendations [14], future costs and health outcomes were both discounted to their present values (in the year 2014) at a rate of 3% per annum. Discounting allows for comparison of costs and benefits across different time periods, by weighting future gains and losses less heavily than those in the present to account for time value.

Both health system (excluding productivity losses) and societal (considering productivity losses and out-of-pocket expenses) perspectives are shown.

Figures are reported to two or three significant figures for visual clarity. Some apparent discrepancies may result due to this rounding.

a Base case model assuming serotype replacement and herd protection (both direct and indirect effects of vaccine).

b An alternate conservative model considering direct effects of vaccine alone (no serotype replacement and no herd protection).

**Table 4:** Incremental cost-effectiveness ratios per DALY averted from PCV13 introduction in alternative scenarios to the base case.

	Direct & indirect population effects	
	PCV13 (with serotype replacement and herd protection) vs. no PCV	
	Societal perspective	Health system perspective
Base case		
Base case	Cost-saving	$52

Cost		
*Scenario 1:* Vaccine cost using maximum AMC tail price (US$3.50)	Cost-saving	$58
*Scenario 2:* Vaccine cost using PAHO price (US$15.68)	$390	$460
*Scenario 3:* Hospitalization cost using WHO-CHOICE	Cost-saving	$17

Incidence & case fatality risk (Meningitis, Pneumonia, NPNM IPD)		
*Scenario 4:* Lower bound from regional estimates for Western Pacific [43]	Cost-saving	$180
*Scenario 5:* Upper bound from regional estimates for Western Pacific [43]	Cost-saving	$10
	Direct population effects only	
	PCV13 (no serotype replacement, no herd protection) vs. no PCV	
	Societal perspective	Health system perspective

Base case		
Base case	$19	$79

Cost		
*Scenario 1:* Vaccine cost using maximum AMC tail price (US$3.50)	$26	$87
*Scenario 2:* Vaccine cost using PAHO price (US$15.68)	$480	$540
*Scenario 3:* Hospitalization cost using WHO-CHOICE	Cost-saving	$27

Incidence & case fatality risk (Meningitis, Pneumonia, NPNM IPD)		
*Scenario 4:* Lower bound from regional estimates for Western Pacific [43]	$110	$312
*Scenario 5:* Upper bound from regional estimates for Western Pacific [43]	Cost-saving	$46

Figures are reported to two significant figures for visual clarity.
