# Supplementary material for: Cost-effectiveness of 13-valent pneumococcal conjugate vaccination in Mongolia
Source: Vaccine. 2017 Feb 15;35(7):1055–63. doi: 10.1016/j.vaccine.2016.12.070 (PMC5297341; doi:10.1016/j.vaccine.2016.12.070)
Supplement: Supplementary data 1 [file mmc1.docx]

Supplementary material

Parameter estimation details, tornado diagram and country profiles

Article title: Cost-effectiveness of 13-valent pneumococcal conjugate vaccination in Mongolia

Authors: Neisha Sundaram, Cynthia Chen, Joanne Yoong, Munkh-Erdene Luvsan, Kimberley Fox, Amarzaya Sarankhuu, Sophie LaVincente, Mark Jit

1. Disease burden and cost parameter estimation details
   1. Disease burden

Incidence and case-fatality risks (CFRs) and of pneumococcal meningitis, non-pneumonia non-meningitis (NPNM) invasive pneumococcal disease (IPD) and pneumonia was obtained from official 2009 WHO estimates for Mongolia among children under five years of age for the year 2000[[1](#_ENREF_1)]. An incidence of 11 per 100,000 for pneumococcal meningitis was estimated in the absence of vaccination. This was similar to that reported (11 per 100,000) in a study that examined childhood pneumococcal meningitis in Ulaanbaatar[[2](#_ENREF_2)]. The WHO incidence estimate for NPNM IPD was 62 per 100,000. Due to a lack of local age-specific data, for meningitis and NPNM IPD the Mongolia incidence was scaled by the age-specific incidences reported in the PneumoNet study by Capeding et al. based on hospitalized children under five in three sites in the Philippines[[3](#_ENREF_3)]. Incidence of both was assumed to be zero for individuals five years or greater.

The WHO estimate for pneumococcal pneumonia incidence was 1345 per 100,000[[1](#_ENREF_1)]. Again, age-specific incidences for Mongolia were estimated due to a lack of local data, in this case by applying age-specific scale factors derived from a study of all-cause radiologic pneumonia in rural Thailand in 2005-2010 for children under 5[[4](#_ENREF_4)] and in 2008-2010 for older ages (Kip Baggett, personal communication) to the overall incidence estimate. For proportion of pneumonia that is invasive vs non-invasive, a 2010 systematic review of childhood pneumonia from 192 countries estimated that 30% of pneumonia cases due to *S. pneumoniae* in Mongolia were severe (hospitalized) [[5](#_ENREF_5)]. Assuming that 10% of all hospitalized pneumococcal pneumonia is invasive and that all invasive cases are hospitalized, we estimate that 3% of all pneumococcal pneumonia was invasive.

CFR estimates for pneumococcal meningitis, NPNM IPD and pneumonia were 34.6%, 27.1% and 5.4%, respectively among children under 5[[1](#_ENREF_1)]. The CFR estimate for pneumonia was applied to all ages.

Acute otitis media (AOM) incidence was obtained from a systematic review of published studies[[6](#_ENREF_6)]. Age-specific incidences for East Asia were used for Mongolia.

- 1. Health care utilisation and costs

All AOM cases presenting to health care were assumed to be treated as outpatients while more serious pneumonia, meningitis, and NPNM IPD were assumed to be hospital inpatient cases. The percentage of medical care-seeking for children with pneumonia symptoms (87%) was obtained from the 2010 Mongolia Multiple Indicator Cluster Survey[[7](#_ENREF_7)]. For AOM, studies from high income countries suggest that 70-100% of children seek medical care. This indicator and the next best proxy, the proportion of febrile children under 5 taken for medical care, are not available for Mongolia. Hence we used 48%, the proportion of children under 5 with acute respiratory illness or fever taken to a health care provider from the Kazakhstan Demographic and Health Survey[[8](#_ENREF_8)].

The 2012 average cost per hospitalized case of meningitis was obtained through data from Mongolia’s health insurance fund and estimated as $217 (MNT 391,620). The same cost was assumed for NPNM IPD. Hospitalization costs for an episode of pneumonia were obtained from a 2012 pneumonia costing study in Mongolia. Data for the pneumonia costing study was collected across six hospitals in Mongolia: three in Ulaanbaatar and one hospital each in provinces of Khovd, Dornod and Orkhon. Unpublished report communicated by health economist from the National Center for Communicable Diseases, Mongolia.Finally, for outpatient AOM treatment cost we use the per-visit payment for outpatient treatment as determined in 2014 by MOH and Social Health Insurance Office.

- 1. Other societal costs

The number of lost work days due to an episode of pneumococcal disease was estimated using the average hospital length of stay for each condition, based on 2012 hospitalization records matched by ICD-10 codes from the National Center for Health Development. The median lengths of stay for meningitis, sepsis (used as a proxy for NPNM IPD) and pneumonia were 10, 9 and 7 days, respectively. For AOM, the number of days of work lost was assumed to be one (based on the assumption of a half-day diagnostic visit and a half-day follow-up visit required). To calculate productivity losses for caregivers of children under 18 years old, the average hospital stay or health centre visit was multiplied by the female labour force participation rate (58.4%)[[9](#_ENREF_9)]. Each day of work lost was valued based on Mongolia’s GDP per capita[[10](#_ENREF_10)].

The WHO global health expenditure database estimates out-of-pocket (OOP) expenditures for Mongolia in 2012 as 35% of total health expenditure (THE)[[11](#_ENREF_11)]. Hospitalization or health centre costs for meningitis, NPNM IPD and AOM were scaled down by a factor of 0.35/0.65 to estimate OOP expenses. OOP expenses per episode of pneumonia were derived through surveys with patients and medical records from the pneumonia costing study^3^ ($69.8), which is very close to the estimate derived from scaling down hospitalization costs for pneumonia ($69.1).

- 1. Disability weights

Disability-adjusted life years (DALYs) lost due to non-fatal pneumococcal meningitis, pneumonia, NPNM IPD and AOM were respectively obtained from disability weights per episode for meningitis due to *S. pneumoniae*, neonatal pneumonia, meningococcaemia without meningitis and otitis media in the 2001 update to the WHO’s Global Burden of Disease[[12](#_ENREF_12)]. The risk of different kinds of major sequelae of pneumococcal meningitis was obtained from a global meta-analysis[[13](#_ENREF_13)], adjusted downwards by the ratio of the study’s estimates of the risk of major sequelae in the WHO Western Pacific region (WPR, 15%) to the global figure (18.6%). The risk of each kind of sequelae was then multiplied by the DALY loss for a year with the corresponding chronic condition (Table 1) to obtain the overall DALY loss for meningitis sequelae.

1. Tornado diagram


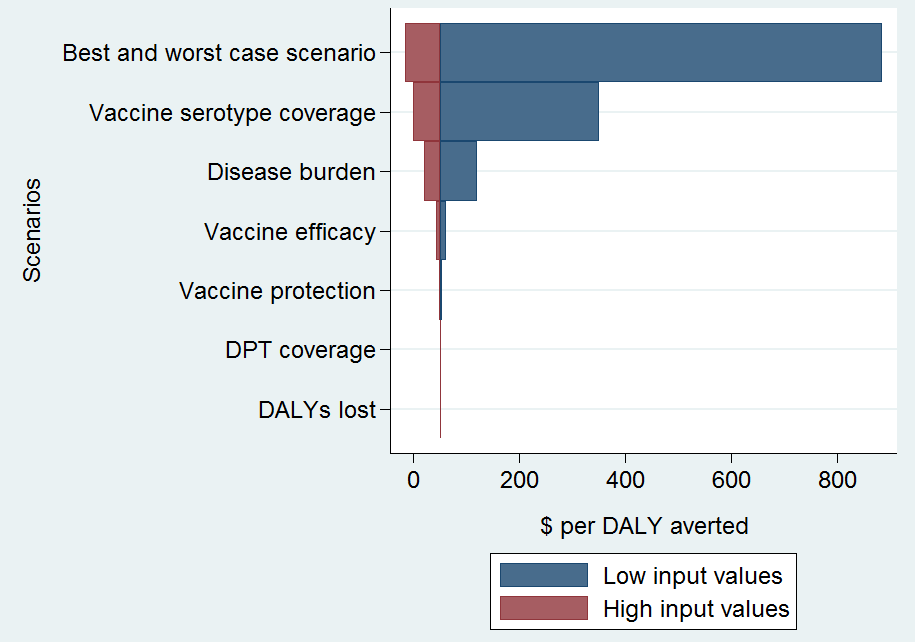


**Supplementary figure.** Tornado diagram summarizing one-way sensitivity analyses varying vaccine-related parameters, disease burden and disability weights by ±25%

*Diagram is centred at the base case scenario that considers direct and indirect population effects from a health system perspective. Longer bars represent assumptions to which the model is more sensitive. Input parameters listed on the left of the graph were varied by low (-25%) and high (+25%) input values to consider the effect on incremental cost-effectiveness ratios. A ‘worst’ case scenario displays the effect of varying all parameters listed in the diagram to their least favourable values and similarly ‘best’ case scenario displays the effect of varying all parameters listed in the diagram to their most favourable values. DALYs: disability-adjusted life years*

1. Country profiles

Supplementary table. Country profiles

| **Parameter** | **Mongolia** | **Philippines** | **Thailand** |
| --- | --- | --- | --- |
| Population median age (years, 2013) | 26.8^a^ | 23^a^ | 36.9^a^ |
| Population aged under 15 (%, 2013) | 27^b^ | 34^c^ | 18^d^ |
| Life expectancy at birth male/female (years, 2013) | 64/72^e^ | 65/72^f^ | 71/79^g^ |
| Gross national income per capita (PPP international $, 2013) | 8^e^ | 7^f^ | 13^g^ |
| Total expenditure on health per capita (international $, 2013) | 567^e^ | 287^f^ | 658^g^ |
| ARI as a causes of death in children under 5 (% of total, 2013) | 16^b^ | 17^c^ | 8^d^ |
| Neonatal sepsis as a causes of death in children under 5 (% of total, 2013) | 5^b^ | 7^c^ | 5^d^ |
| Children aged <5 years with ARI symptoms taken to a health facility (%, 2013) | 70.3^a^ | 64^a^ | 83.3^a^ |

%: percentage; $: dollar; PPP: purchasing power parity; ARI: acute respiratory infection

^a^ WHO Global Health Observatory. http://www.who.int/gho/database/en/. Accessed 1-May-2016

^b^ Mongolia: WHO statistical profile. http://www.who.int/gho/countries/mng.pdf?ua=1. Accessed 1-May-2016

^c^ Philippines: WHO statistical profile. http://www.who.int/gho/countries/phl.pdf?ua=1. Accessed 1-May-2016

^d^ Thailand: WHO statistical profile. http://www.who.int/gho/countries/tha.pdf?ua=1. Accessed 1-May-2016

^e^ WHO country, Mongolia. http://www.who.int/countries/mng/en/. Accessed 1-May-2016

^f^ WHO country, Philippines. http://www.who.int/countries/phl/en/. Accessed 1-May-2016

^g^ WHO country, Thailand. http://www.who.int/countries/tha/en/. Accessed 1-May-2016

References

[1] World Health Organization. Letter from EPI Coordinator: Estimates of severe illness cases and deaths in children from one month to less than five years of age due to *Streptococcus pneumoniae* in Mongolia. 2009.

[2] Mendsaikhan J, Watt JP, Mansoor O, Suvdmaa N, Edmond K, Litt DJ, et al. Childhood bacterial meningitis in Ulaanbaatar, Mongolia, 2002-2004. Clin Infect Dis. 2009;48 Suppl 2:S141-S6.

[3] Capeding MR, Bravo L, Santos J, Kilgore PE, Kim SA, Balter I, et al. Prospective surveillance study of invasive pneumococcal disease among urban children in the Philippines. Pediatr Infect Dis J. 2013;32:e383-e9.

[4] Hasan R, Rhodes J, Thamthitiwat S, Olsen SJ, Prapasiri P, Naorat S, et al. Incidence and etiology of acute lower respiratory tract infections in hospitalized children younger than 5 years in rural Thailand. Pediatr Infect Dis J. 2014;33:e45-e52.

[5] Rudan I, O'Brien KL, Nair H, Liu L, Theodoratou E, Qazi S, et al. Epidemiology and etiology of childhood pneumonia in 2010: estimates of incidence, severe morbidity, mortality, underlying risk factors and causative pathogens for 192 countries. J Glob Health. 2013;3:010401.

[6] Monasta L, Ronfani L, Marchetti F, Montico M, Vecchi Brumatti L, Bavcar A, et al. Burden of disease caused by otitis media: systematic review and global estimates. PloS one. 2012;7:e36226.

[7] National Statistics Office, UNICEF. "Multiple Indicator Cluster Survey 2010" Summary Report. Ulaanbaatar, Mongolia: 2011.

[8] Academy of Preventive Medicine [Kazakhstan], Macro International Inc. Kazakhstan Demographic and Health Survey 1999. Calverton, Maryland: Academy of Preventive Medicine and Macro International Inc.; 1999.

[9] National Statistical Office of Mongolia. Statistical Yearbook - 2012. Ulaanbaatar: National Statistical Office; 2013.

[10] World Bank. World Development Indicators, GDP per capita (current US$) for Mongolia. <http://data.worldbank.org/data-catalog/world-development-indicators> (accessed 3/11/2014).

[11] World Health Organization. Global Health Expenditure Database. <http://apps.who.int/nha/database/Select/Indicators/en> (accessed 3/10/2015).

[12] Mathers CD, Lopez AD, Murray CJL. The Burden of Disease and Mortality by Condition: Data, Methods, and Results for 2001. In: Lopez AD, Mathers CD, Ezzati M, Jamison DT, Murray CJL, editors. Global Burden of Disease and Risk Factors. Washington (DC): World Bank; 2006.

[13] Edmond K, Clark A, Korczak VS, Sanderson C, Griffiths UK, Rudan I. Global and regional risk of disabling sequelae from bacterial meningitis: a systematic review and meta-analysis. The Lancet Infectious diseases. 2010;10:317-28.
